# Self-perceptions of aging and associated factors among older patients undergoing maintenance hemodialysis: a latent profile analysis

**DOI:** 10.3389/fpubh.2026.1832983

**Published:** 2026-05-22

**Authors:** Weiwei Yang, Chengxin Fu, Yang Liu, Huaihong Yuan

**Affiliations:** 1 Department of Nephrology, Institute of Kidney Diseases, West China Hospital of Sichuan University, Chengdu, China; 2 West China School of Nursing, Sichuan University, Chengdu, China

**Keywords:** depression, latent profile analysis, maintenance hemodialysis, older adults, self-perceptions of aging, social frailty

## Abstract

**Background:**

Self-perceptions of aging (SPA) were significantly associated with adverse health outcomes in older adults. However, whether SPA among older patients undergoing maintenance hemodialysis (MHD) exhibits distinct latent profiles and what variables are associated with these subgroups remain to be clarified.

**Objective:**

This study aimed to identify the latent profiles of SPA and examine their related factors among older MHD patients.

**Methods:**

A secondary analysis was carried out on a cross-sectional study. A convenience sampling method was employed to recruit older MHD patients from four hemodialysis centers in Sichuan Province, China. Data were collected using a demographic information form, the Brief Aging Perceptions Questionnaire (B-APQ), the Social Frailty Scale (SFS), and the 5-item Geriatric Depression Scale (GDS-5). Latent profile analysis was employed to identify distinct subgroups of SPA, and binary logistic regression was adopted to explore factors associated with subgroup membership.

**Results:**

A total of 381 older MHD patients were included in this study. Two latent profiles were identified and were designated as the “positive self-perceptions of aging group (36.5%)” and “negative self-perceptions of aging group (63.5%).” Age ≤ 70 years was negatively associated with the negative self-perceptions of aging group (OR = 0.56, 95% CI: 0.34–0.93, *p* = 0.024), while depression (OR = 1.96, 95% CI: 1.15–3.32, *p* = 0.013) and social frailty (OR = 3.21, 95% CI: 2.00–5.15, *p* < 0.001) were positively associated with that group.

**Conclusion:**

A multi-level support system involving healthcare providers, family members, and community resources should be established to address the psychological needs of older MHD patients and mitigate negative perceptions of aging.

## 1 Introduction

End-stage renal disease (ESRD) constitutes a growing global health concern owing to its expanding prevalence, substantial costs, and irreversible progression (1–3). Maintenance hemodialysis (MHD) remains the primary treatment for ESRD. By removing toxins, balancing electrolytes and controlling fluid levels, it enables patients to survive (4). However, the efficacy of MHD and its complications vary significantly across different age groups, with older patients facing greater clinical complexity than younger patients. Specifically, older MHD patients generally experience a more extensive symptom burden, such as fatigue, sleep disorders, and frailty, along with more severe psychological distress, including depression and anxiety (5–8). These situations highlight the urgent necessity of taking this population into consideration in public health policy.

Self-perceptions of aging (SPA) refer to an individual’s subjective view of their own aging process, encompassing physiological, psychological, and social domains (9). It has been demonstrated that negative SPA is significantly associated with adverse outcomes such as cognitive decline, impaired social functioning, and mortality rates among older adults (10–12). In contrast, positive SPA plays a vital role in successful aging by encouraging adaptive coping strategies and behaviors that promote health (13–15). SPA takes on particular significance in the context of older MHD patients. For them, the experience of aging is accelerated and intensified by the dual burden of ESRD and its rigorous hemodialysis treatment (16). The relentless cycle of dialysis dependency, coupled with pervasive symptoms such as fatigue, pain, frailty, and functional limitations, acts as a constant and powerful reminder of physiological decline (17). This unique clinical scenario creates fertile ground for negative aging beliefs, which may contribute to a heightened sense of vulnerability and diminished self-worth. Despite this vulnerability, the patterns and heterogeneity of SPA within this population have not been sufficiently explored.

Prior research on SPA has mainly relied on scale total scores to determine the level of SPA and has explored its associations with depression among community-dwelling older adults and with daily living ability among empty nesters (18, 19). While informative, this variable-centered approach inevitably overlooks population heterogeneity, as individuals with identical total scores may exhibit markedly different response patterns across specific items. Consequently, conclusions drawn from such analyses may not fully capture the actual state of SPA. Latent profile analysis (LPA), a person-centered approach widely adopted in psychological and medical research, addresses this methodological limitation by identifying unobserved subgroups that share similar response profiles (20, 21). In the hemodialysis context, a growing body of cross-sectional work employing conventional regression approaches has identified several correlates of attitudes toward aging in older MHD patients, including sex, interdialytic weight gain, number of comorbidities, self-regulatory fatigue, treatment adherence, and social participation (16, 22). However, the potential roles of depression and social frailty, two clinically relevant psychological constructs in this population, have yet to be examined in relation to SPA. The self-regulation model offers a useful framework for understanding these relationships, positing that individuals interpret health threats such as chronic illness through their perceptions of consequences, personal control, timeline, and emotional responses, and that these perceptions are associated with how they evaluate their own aging process (23). From this perspective, depressive symptoms in older MHD patients may be associated with more negative emotional representations and perceived consequences of aging (24), while social frailty may be linked to such perceptions through diminished social support and reduced participation (25). Building on these theoretical and empirical grounds, the present study sought to identify latent profiles of SPA among older MHD patients using LPA and to examine the associations of these profiles with depression and social frailty.

## 2 Materials and methods

### 2.1 Study design, setting, and participants

This study was a secondary analysis of data from a previous cross-sectional study (26). While the original study focused on social frailty and its related factors (family function, self-care ability, depression, and physical frailty), this study employed LPA to identify distinct latent profiles of SPA and used logistic regression to explore the associations of demographic characteristics, depression, and social frailty with these profiles. Older MHD patients were recruited through convenience sampling at four hemodialysis centers in Sichuan Province, China, between September and December 2024. The inclusion criteria included: (1) aged ≥ 60; (2) diagnosed with ESRD and receiving MHD treatment for at least 3 months; (3) ability to communicate verbally; (4) willingness to participate in the survey. The exclusion criteria were as follows: (1) presence of cognitive impairment or severe mental illness; (2) physical frailty precluding completion of the survey; (3) significant visual or hearing impairment.

The sample size was determined following Kendall’s guideline for cross-sectional studies (27), which recommends a minimum of 10 participants per independent variable with an additional 10–20% allowance for potential invalid responses. Based on the nine key variables examined in this study, the minimum required sample size was calculated as 99 participants. From the initial distribution of 402 questionnaires, 381 valid responses were obtained, achieving a high response rate of 94.8%.

### 2.2 Measurements

#### 2.2.1 Demographic information form

The structured questionnaire consists of seven demographic variables: gender, age, educational background, marital status, monthly personal income, residence, and hemodialysis duration.

#### 2.2.2 Brief Aging Perceptions Questionnaire (B-APQ)

The B-APQ was initially developed by Sexton et al. (28) based on the self-regulation model, and was later adapted into Chinese by Hu et al. (29). This 17-item instrument assesses the SPA across five distinct dimensions: timeline-chronic, consequences-positive, control-positive, consequences and control negative, and emotional representations. Responses were recorded on a 5-point Likert scale (1 = strongly disagree, 5 = strongly agree), with items 4–6 and 8–10 reverse-scored to mitigate response bias. Total scores ranged from 17 to 85, with higher values indicating a more negative SPA. A prior study has shown that the Chinese version exhibits excellent reliability (Cronbach’s *α* = 0.914) (29), and our analysis further confirmed its robust internal consistency (Cronbach’s *α* = 0.942).

#### 2.2.3 Social Frailty Scale (SFS)

Social frailty among older MHD patients was assessed using the SFS developed by Makizako (30). This validated instrument evaluates social frailty through five criteria: (1) living alone; (2) infrequent social interactions (absence of daily conversations); (3) decreased frequency of outings compared to the previous year; (4) self-perceived inability to provide instrumental support to family/friends; (5) limited social visits (rarely seeing friends). Participants meeting ≥ 2 criteria were classified as social frailty, consistent with the original scale’s operational definition. In the present study, the SFS demonstrated acceptable internal consistency (Cronbach’s *α* = 0.761).

#### 2.2.4 5-item Geriatric Depression Scale (GDS-5)

The GDS-5, compiled by Hyol, is an updated version of the original 15-item Geriatric Depression Scale (GDS-15), with excellent demonstrated validity and reliability (31). This instrument comprises five items with binary response options (Yes or No), yielding a total score ranging from 0 to 5. Any score of 2 or higher indicates the presence of clinically significant depressive symptoms. In the present study, the GDS-5 exhibited acceptable internal consistency, with a Cronbach’s *α* of 0.768.

### 2.3 Data collection

Data collection was conducted via the Questionnaire Star platform between September and December 2024. Eight trained registered nurses served as investigators, participating in and taking responsibility for the entire data collection process. Before survey administration, all participants provided informed consent after receiving detailed explanations regarding the purpose, significance, procedures, and ethical considerations in this study. All participants were provided with a QR code to access the survey during their waiting interval for dialysis at the outpatient department. To ensure data quality, some rigorous quality control measures were employed: (1) temporal validation by excluding questionnaires completed in < 10 or > 20 min; (2) response pattern screening to detect uniform or regular answering tendencies; (3) logical consistency verification checks. All questionnaire items were mandatory and had undergone pretesting for clarity.

### 2.4 Data analysis

Latent profile analysis was conducted using Mplus 8.3 to identify distinct latent profiles based on the item scores of the B-APQ. The number of subgroups was selected by the fit indices resulting from the increasing categorical quantities. The model fit was assessed using multiple established criteria: (1) information criteria including the Akaike information criterion (AIC), Bayesian information criterion (BIC), and adjusted BIC (aBIC), where lower values represent better model fit; (2) comparative fit tests comprising the Lo–Mendell–Rubin likelihood ratio test (LMRT) and bootstrapped likelihood ratio test (BLRT), with statistically significant results (*p* < 0.05) indicating that the k-class solution provided significantly better fit than the k-1 class solution; (3) entropy values ranging from 0 to 1, with higher values (closer to 1) reflecting greater classification accuracy of the latent profile solution.

Statistical analyses were conducted using SPSS 27.0. The Shapiro–Wilk test was used to assess the normality of the total and dimension scores of SPA. Categorical variables were described by counts and proportions, and normally distributed continuous variables were expressed as mean and standard deviation. Furthermore, comparative analyses between groups were performed utilizing the chi-square tests. Binary logistic regression analysis was employed to explore the variables associated with SPA among older MHD patients. A two-sided *p* < 0.05 was considered statistically significant. A sensitivity analysis was conducted using the R3STEP procedure in Mplus 8.3, with all covariates simultaneously entered as auxiliary variables to account for classification uncertainty.

## 3 Results

### 3.1 Characteristics and B-APQ scores of participants

A total of 381 older patients with MHD were included in this study (Table 1). The majority of participants were male (57.5%), and 64.0% were aged 70 years or younger. Educational background was variably distributed, with 42.5% of participants having attained senior high school level or above. In addition, most participants were married (85.3%), and 42.0% reported a personal monthly income of less than 3,000 RMB. Furthermore, the sample was predominantly urban (81.9%). In terms of clinical characteristics, 56.2% had been on MHD for less than 5 years. Moreover, depression was identified in 33.9% of participants, while social frailty was present in 55.9%.

**Table 1:** Characteristics and B-APQ scores among older MHD patients.

Variables	N/(M ± SD)	Percent
Gender		
Male	219	57.5%
Female	162	42.5%
Age (years)		
≤ 70	244	64.0%
> 70	137	36.0%
Educational background		
Primary school or lower	128	33.6%
Junior high school	91	23.9%
Senior high school	75	19.7%
University or above	87	22.8%
Marital status		
Unmarried	3	0.8%
Married	325	85.3%
Divorced/widowed	53	13.9%
Personal monthly income (RMB)		
< 3,000	160	42.0%
3,000–5,000	106	27.8%
> 5,000	115	30.2%
Residence		
Urban	312	81.9%
Rural	69	18.1%
Hemodialysis duration (years)		
< 5	214	56.2%
5–10	94	24.7%
> 10	73	19.1%
Depression		
Yes	129	33.9%
No	252	66.1%
Social frailty		
Yes	213	55.9%
No	168	44.1%
B-APQ	50.1 ± 7.7	/
Timeline-chronic	10.1 ± 2.6	/
Consequences-positive	8.0 ± 1.9	/
Control-positive	6.6 ± 1.8	/
Consequences and control negative	17.1 ± 3.6	/
Emotional representations	8.3 ± 2.3	/

The Shapiro–Wilk test indicated that the total B-APQ score and its five dimensions scores were all normally distributed (all *p* > 0.05). The total B-APQ score was 50.1 ± 7.7, and the dimension scores were timeline-chronic (10.1 ± 2.6), consequences-positive (8.0 ± 1.9), control-positive (6.6 ± 1.8), consequences and control negative (17.1 ± 3.6), and emotional representations (8.3 ± 2.3) (Table 1).

### 3.2 Latent profile determination

**Figure 1:**
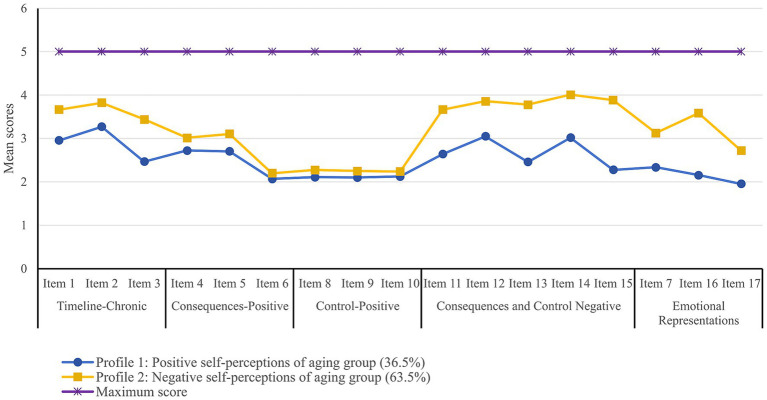
Two latent profiles of SPA among older MHD patients.

According to the results of the SPA assessment among older MHD patients, three latent profile models were established (Table 2). As the number of profiles increased, the AIC, BIC, and aBIC values declined. Although the three-profile solution yielded more favorable AIC, BIC, aBIC, and entropy values, along with a significant BLRT (*p* < 0.001), its LMRT did not reach statistical significance (*p* = 0.0945), indicating no meaningful improvement in fit over the two-profile model. Furthermore, inspection of the three-profile plot (Supplementary Figure S1) revealed that Profile 3 (15.2%) closely resembled Profile 2 on core negative dimensions, with the separation driven primarily by a single dimension (control-positive). On the remaining core negative dimensions, Profile 3 did not depart substantively from the configural pattern of Profile 2, and thus failed to constitute a coherent, dimensionally distinct aging perception profile. This limited its substantive interpretability and offered negligible additional value for discriminative or clinical targeting. Given the non-significant LMRT and the localized nature of the differentiation, the two-profile model provided clearer clinical separation with fewer parameters. Balancing statistical parsimony with the feasibility of clinical intervention, we retained the two-profile model as the final solution. The 17-item scores corresponding to the two latent profiles of SPA among older MHD patients are detailed in Figure 1. Profile 1 was defined by lower scores on each item, indicating less negative perceptions of aging and a more positive SPA profile, and was therefore designated as the “positive self-perceptions of aging group” (36.5%). In contrast, Profile 2 exhibited consistently higher scores across all 17 items compared to Profile 1, indicating a more negative attitude towards aging. Consequently, it was named the “negative self-perceptions of aging group” (63.5%).

**Table 2:** Indicators for each latent profile of SPA among older MHD patients.

Model	AIC	BIC	aBIC	Entropy	LMRT (*P*)	BLRT (*P*)	Categorical probability
1	16859.455	16993.511	16885.635				
2	15898.505	16103.531	15938.544	0.890	< 0.001	< 0.001	0.365/0.635
3	15364.776	15640.771	15418.674	0.926	0.0945	< 0.001	0.336/0.512/0.152

AIC, Akaike information criterion; BIC, Bayesian information criterion; aBIC, Adjusted Bayesian information criterion; LMRT, Lo–Mendell–Rubin likelihood ratio test; BLRT, Bootstrapped likelihood ratio test.

### 3.3 Comparison of characteristics of participants in different latent profiles

The results of the univariate analysis targeting two SPA profiles are outlined in Table 3. A comparison of SPA characteristics among older MHD patients of various ages, educational backgrounds, personal monthly incomes, residences, depression, and social frailty demonstrated statistically significant differences (all *p* < 0.05).

**Table 3:** Univariate analysis of latent profiles of SPA among older MHD patients.

Variables	Profile 1 (*n* = 139)	Profile 2 (*n* = 242)	*χ* ^2^	*p*
Gender			0.119	0.730
Male	82 (59.0%)	137 (56.6%)		
Female	57 (41.0%)	105 (43.4%)		
Age (years)			8.940	0.003
≤ 70	103 (74.1%)	141 (58.3%)		
> 70	36 (25.9%)	101 (41.7%)		
Educational background			13.205	0.004
Primary school or lower	32 (23.0%)	96 (39.7%)		
Junior high school	33 (23.7%)	58 (24.0%)		
Senior high school	34 (24.5%)	41 (16.9%)		
University or above	40 (28.8%)	47 (19.4%)		
Marital status			3.831	0.147
Unmarried	1 (0.7%)	2 (0.8%)		
Married	125 (89.9%)	200 (82.7%)		
Divorced/widowed	13 (9.4%)	40 (16.5%)		
Personal monthly income (RMB)			9.621	0.008
< 3,000	45 (32.4%)	115 (47.5%)		
3,000–5,000	41 (29.5%)	65 (26.9%)		
> 5,000	53 (38.1%)	62 (25.6%)		
Residence			4.497	0.034
Urban	122 (87.8%)	190 (78.5%)		
Rural	17 (12.2%)	52 (21.5%)		
Hemodialysis duration (years)			0.800	0.670
< 5	74 (53.2%)	140 (57.8%)		
5–10	36 (25.9%)	58 (24.0%)		
> 10	29 (20.9%)	44 (18.2%)		
Depression			17.428	< 0.001
Yes	28 (20.1%)	101 (41.7%)		
No	111 (79.9%)	141 (58.3%)		
Social frailty			39.199	< 0.001
Yes	48 (34.5%)	165 (68.2%)		
No	91 (65.5%)	77 (31.8%)		

### 3.4 Binary logistic regression of latent profiles of self-perceptions of aging

A binary logistic regression analysis was employed to examine the variables associated with SPA profiles among older MHD patients (Table 4). Variables statistically significant in the univariate analysis were entered as independent variables, with SPA profile as the dependent variable. All multi-categorical variables were converted into dummy variables, using the last category as the reference group. With Profile 1 as the reference, age ≤ 70 years (OR = 0.557; *p* = 0.024), depression (OR = 1.955; *p* = 0.013), and social frailty (OR = 3.213; *p* < 0.001) were significantly associated with Profile 2. The R3STEP sensitivity analysis, which accounts for classification uncertainty, confirmed the significance and direction of these associations (Supplementary Table S1). All covariates were entered simultaneously, yielding a total sample of 381 participants with complete data on all covariates.

**Table 4:** Binary logistic regression of SPA profiles among older MHD patients (Ref: Profile 1).

Variables	*B*	SE	Wald	*p*	OR	95% CI
Age						
≤ 70	−0.585	0.260	5.068	0.024	0.557	0.335–0.927
> 70	Ref					
Educational background						
Primary school or lower	0.506	0.423	1.426	0.232	1.658	0.723–3.802
Junior high school	0.116	0.382	0.093	0.761	1.123	0.531–2.375
Senior high school	−0.183	0.373	0.241	0.624	0.833	0.401–1.730
University or above	Ref					
Personal monthly income						
< 3,000	0.424	0.374	1.281	0.258	1.527	0.733–3.181
3,000–5,000	0.332	0.330	1.010	0.315	1.394	0.729–2.663
> 5,000	Ref					
Residence						
Urban	−0.033	0.387	0.007	0.931	0.967	0.453–2.065
Rural	Ref					
Depression						
Yes	0.670	0.270	6.148	0.013	1.955	1.151–3.321
No	Ref					
Social frailty						
Yes	1.167	0.241	23.446	< 0.001	3.213	2.003–5.154
No	Ref					

## 4 Discussion

By applying a person-centered LPA approach to identify latent profiles of SPA among older MHD patients, this study extends prior research that has primarily relied on total scale scores. Unlike conventional regression analyses that assume population homogeneity and may overlook heterogeneity in SPA patterns, this approach revealed that older MHD patients could be classified into distinct SPA profiles. These findings add to existing knowledge and may help guide the development of targeted interventions aimed at improving psychological well-being in this population.

This study identified two latent profiles of SPA in older MHD patients: “positive self-perceptions of aging group (36.5%)” and “negative self-perceptions of aging group (63.5%).” With nearly two-thirds of patients classified into the negative SPA group, these results highlighted the predominance of negative SPA in this population. Several factors may be relevant to these findings, spanning physiological, psychological, and social domains. To begin with, older MHD patients were more likely to experience complications such as fatigue, itching, and sarcopenia (5, 32, 33), and these complications were related to greater perceived physical frailty and a more negative sense of aging. In addition, previous studies have reported a high prevalence of depression and anxiety among older MHD patients, conditions that have been tied to cognitive biases toward negative self-perceptions (34). Finally, demanding dialysis schedules often coincide with limited opportunities for social role engagement in this population, and the loneliness and social isolation that accompany these constraints are also associated with more negative SPA (35). Given these findings, healthcare professionals should prioritize SPA screening for older MHD patients and provide appropriate and accessible education to support their understanding of the aging process, thereby promoting their overall quality of life.

Our findings indicated that older MHD patients aged ≤ 70 years were less likely to be classified into the “negative self-perceptions of aging group” than those aged > 70 years. Those aged ≤ 70 years are often described as “young seniors” (36), many of whom maintain a relatively strong sense of vitality and remain highly engaged in social and family life (37). Conversely, the greater likelihood of negative SPA in patients aged > 70 years is consistent with the typical life stage transitions, such as withdrawal from core social roles, as well as with expectations of reduced social participation (38). In light of these patterns, tailoring support according to age group may help enhance psychological well-being and improve the treatment experience of older MHD patients.

Our findings identified a strong association between depression and SPA among older MHD patients. Specifically, older MHD patients with depression were at greater risk of being categorized into the “negative self-perceptions of aging group”. Depression is prevalent among this population and is linked to poor mental health outcomes (39). As previous research has indicated, individuals with depression typically exhibit negative cognitive patterns and catastrophic thinking, which are associated with self-depreciation and denial of self-worth (40, 41). Furthermore, the combination of long-term treatment dependence and progressive functional limitations during dialysis may be related to negative feelings toward aging. These findings suggest that depression is an important correlate of SPA among older MHD patients. Healthcare providers should receive training to identify and address age-specific psychological distress by incorporating cognitive-behavioral strategies to help patients adopt a more positive attitude toward aging and cope with the psychological stresses of long-term treatment (42, 43).

We found that older MHD patients with social frailty were particularly vulnerable to being clustered into the “negative self-perceptions of aging group.” Social frailty refers to a decline in the individual’s functioning in terms of social relationships and resources, characterized by a shrinking social network, insufficient support, reduced activity, and increased loneliness (44). According to Self-Categorization Theory, when individuals remain isolated for an extended duration in their environment, they may internalize negative archetypal traits associated with the “infirm group” in their self-perceptions (45). Dialysis-related stressors among older MHD patients, including time-intensive treatment schedules, post-dialysis fatigue, and dietary or fluid restrictions, have been linked to social functional decline, possibly through reducing available time, consuming energy, and limiting social activities (26, 46, 47). Moreover, social frailty has been associated with reduced engagement in diverse social roles, which may limit the cognitive resources necessary to counteract negative aging stereotypes and may be related to an internalized sense of accelerated aging (48). These findings suggest that structured community engagement programs may help facilitate meaningful social interactions between older MHD patients and community members, together with education on available resources to promote social participation (49, 50). Additionally, family-centered educational interventions may help enhance familial communication and emotional support to address patients’ mental needs. Such multilevel strategies may be helpful for strengthening social connectedness and mitigating negative SPA (51).

## 5 Limitations

Several limitations of this study warrant consideration. First, the cross-sectional design precludes the establishment of causal relationships. Second, convenience sampling from only four centers in Sichuan Province limits the generalizability of our findings. Furthermore, selection bias may be introduced because the exclusion criteria and QR-code-based data collection excluded many vulnerable patients (those with cognitive impairment, severe mental illness, or marked physical frailty), who may be more likely to hold negative SPA. This bias may underestimate the prevalence of negative SPA. Third, some clinical covariates (such as comorbidity burden, albumin and hemoglobin levels, dialysis adequacy, and other markers of disease severity) were not collected, which may confound the observed associations. Fourth, although the binary logistic regression using categorical class assignments may be subject to classification uncertainty (52), the R3STEP sensitivity analysis yielded convergent results, supporting the robustness of the reported associations. To address these limitations, future research should consider employing longitudinal designs to establish causal pathways. In addition, sampling strategies with broader inclusion criteria are needed to capture the latent profiles of SPA among older MHD patients. Moreover, incorporating more clinical indicators would reduce potential confounding bias. Finally, classification-error-adjusted methods such as R3STEP should be adopted as the primary analytical strategy in future studies to further enhance the precision of estimates.

## 6 Conclusion

In conclusion, our study identified two categories of SPA among older MHD patients: “positive self-perceptions of aging group” and “negative self-perceptions of aging group.” Moreover, age, depression, and social frailty were detected as significant associated factors of distinct SPA profiles. Healthcare professionals should prioritize the identification of specific types of SPA among older MHD patients and develop tailored interventions to meet their psychological needs and mitigate negative aging perceptions.

## Data Availability

The raw data supporting the conclusions of this article will be made available by the authors, without undue reservation.
