# Supplementary material for: Self-perceptions of aging and associated factors among older patients undergoing maintenance hemodialysis: a latent profile analysis
Source: Front Public Health. 2026 May 22;14:1832983. doi: 10.3389/fpubh.2026.1832983 (PMC13236573; doi:10.3389/fpubh.2026.1832983)
Supplement: Supplementary file 1 [file Table_1.DOCX]

Supplementary Material

# Supplementary Tables

Table S1 R3STEP Analysis of Factors Associated with SPA Profiles among Older MHD Patients (Ref: Profile 1)

| **Variables** | **B** | **SE** | **OR** | ***P*** | **95% CI** |
| --- | --- | --- | --- | --- | --- |
| Gender |  |  |  |  |  |
| Male | 0.144 | 0.284 | 1.155 | 0.612 | 0.662–2.015 |
| Female | Ref |  |  |  |  |
| Age |  |  |  |  |  |
| ≤ 70 | -0.617 | 0.290 | 0.540 | 0.033 | 0.306–0.953 |
| > 70 | Ref |  |  |  |  |
| Educational background |  |  |  |  |  |
| Primary school or lower | 0.607 | 0.471 | 1.834 | 0.197 | 0.729–4.619 |
| Junior high school | 0.166 | 0.427 | 1.181 | 0.697 | 0.511–2.726 |
| Senior high school | -0.177 | 0.429 | 0.837 | 0.679 | 0.361–1.942 |
| University or above | Ref |  |  |  |  |
| Marital status |  |  |  |  |  |
| Unmarried | 0.485 | 1.521 | 1.625 | 0.750 | 0.082–32.014 |
| Married | -0.268 | 0.426 | 0.765 | 0.530 | 0.332–1.763 |
| Divorced/widowed | Ref |  |  |  |  |
| Personal monthly income |  |  |  |  |  |
| < 3000 | 0.470 | 0.441 | 1.601 | 0.286 | 0.674–3.798 |
| 3000–5000 | 0.374 | 0.389 | 1.453 | 0.336 | 0.678–3.116 |
| > 5000 | Ref |  |  |  |  |
| Residence |  |  |  |  |  |
| Urban | -0.064 | 0.427 | 0.938 | 0.880 | 0.406–2.166 |
| Rural | Ref |  |  |  |  |
| Hemodialysis duration (years) |  |  |  |  |  |
| < 5 | 0.047 | 0.337 | 1.048 | 0.889 | 0.541–2.029 |
| 5–10 | 0.010 | 0.384 | 1.011 | 0.978 | 0.476–2.144 |
| > 10 | Ref |  |  |  |  |
| Depression |  |  |  |  |  |
| Yes | 0.754 | 0.297 | 2.125 | 0.011 | 1.188–3.804 |
| No | Ref |  |  |  |  |
| Social frailty |  |  |  |  |  |
| Yes | 1.254 | 0.277 | 3.503 | < 0.001 | 2.036–6.031 |
| No | Ref |  |  |  |  |

# Supplementary Figures


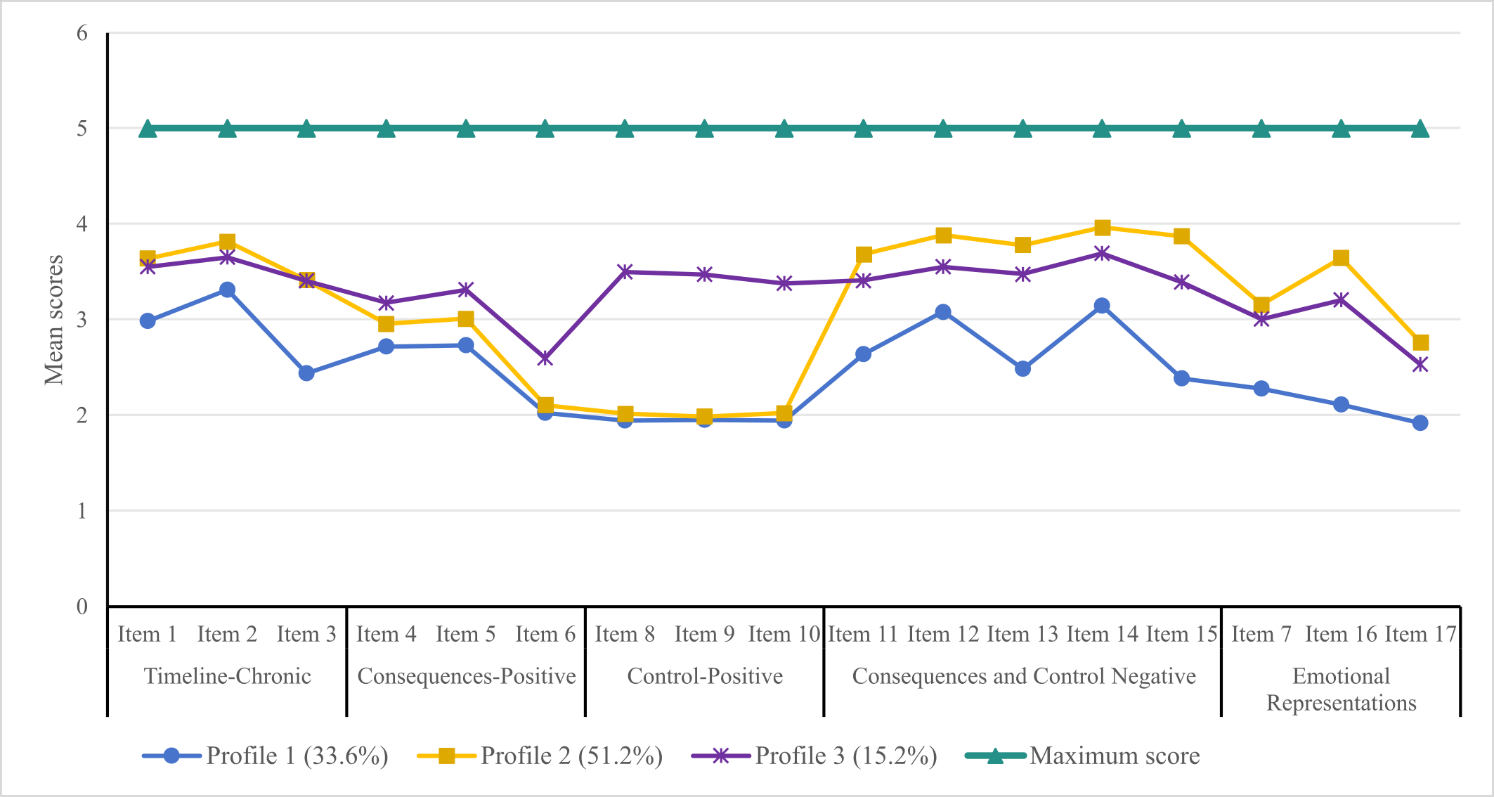


Figure S1 Three Latent Profiles of SPA among Older MHD Patients
